# Antiviral Therapy for Chronic HBV Infection With Persistently Normal Alanine Aminotransferase: Controversy and Consensus

**DOI:** 10.3389/fmed.2021.717125

**Published:** 2021-08-30

**Authors:** Jing Zhou, Fa-Da Wang, Meng-Lan Wang, Ya-Chao Tao, Dong-Bo Wu, Yun-Jian Sheng, Gui-Bao Xiao, Xue-Bin Chen, Xin Chen, En-Qiang Chen

**Affiliations:** ^1^Center of Infectious Diseases, West China Hospital of Sichuan University, Chengdu, China; ^2^Department of Infection, Affiliated Hospital of Southwest Medical University, Luzhou, China; ^3^Department of Infectious Diseases, The First People's Hospital of Ziyang City, Ziyang, China; ^4^Department of Infectious Diseases, People's Hospital of Deyang City, Deyang, China; ^5^Department of Infectious Diseases, The Second People's Hospital of Neijiang, Neijiang, China

**Keywords:** hepatitis B virus infection, normal alanine aminotransferase, immune tolerance, disease progression, antiviral therapy

## Abstract

ALT is one of the most sensitive biochemical indexes to reflect liver injury. It is generally believed that hepatitis B virus (HBV) infected patients with normal ALT levels are in either immune tolerance or low replication stage of the natural history of hepatitis B, and there is no or only mild inflammation in liver tissue, so antiviral therapy is not recommended. However, chronic HBV-infected patients with normal ALT levels are not always in a stable state. A considerable number of patients will develop active hepatitis or occult progress to liver fibrosis, cirrhosis, and hepatocellular carcinoma. Therefore, whether antiviral therapy should be recommended for chronic HBV infection with normal ALT level has been a hot topic in clinical practice. In this paper, the definition of immune tolerance, the relationship between ALT and liver inflammation, and the benefits of antiviral therapy were reviewed, and we hope it will be helpful for clinicians to have a deeper understanding of whether antiviral therapy should be considered for chronic HBV infection with normal ALT.

## Introduction

Hepatitis B virus (HBV) infection is one of the most common infectious diseases in the world. The persistence and active replication of HBV in liver tissue is the main cause of chronic hepatitis, cirrhosis, and hepatocellular carcinoma(HCC). Timely and effective antiviral treatment is the most effective measure to delay and prevent the progress or aggravation of diseases (1). The occurrence of liver damage and its severity depends on the interaction between HBV and host immune system (2). The host immune response can not only clear the virus but also lead to liver inflammation (2, 3).

In East Asian countries, the main route of chronic HBV infection is mother-to-child transmission or infection during infancy. Due to immune tolerance of the host against HBV, some patients are with normal ALT, positive HBeAg, and high HBV DNA levels (4). At present, for such a group of patients, antiviral therapy is not recommended by guidelines unless the patient has evidence of liver injury (5–8).

ALT is the most direct, sensitive, and economic indicator of liver inflammation, and its elevated level usually indicates the occurrence of liver inflammatory (9). Therefore, in addition to positive HBV DNA level, abnormal ALT is also required if antiviral therapy is considered (6, 7). However, ALT in the normal range does not absolutely indicate no inflammatory in liver. Thus, patients with normal ALT is not always in a stable state, and a considerable number of them will develop active hepatitis or occult progression to liver fibrosis, cirrhosis, and HCC (10, 11). Therefore, whether antiviral therapy should be recommended for patients with normal ALT has been a hot topic at present. This article will review the definition of immune tolerance, relationship between ALT and liver inflammation, and benefits of antiviral therapy, so as to help clinicians to have a deeper understanding of whether antiviral therapy should be considered for chronic HBV infection with normal ALT.

## Definition and Connotation of Immune Tolerance

### What Is Immune Tolerance?

Generally, immune tolerance is defined as the specific non-response state of the immune system after receiving specific antigen. The liver has a unique immune regulation function, which can promote the tolerance to HBV, and this may be the main reason of HBV persistence and chronic infection. In 1972, Dudley et al. firstly proposed that HBV persistent infection was related to immune tolerance, and the liver injury was determined by T cell-mediated immune response (12). In 1983, Liaw et al. reported that HBeAg clearance was related to the enhancement of host immune response (13). Based on the above findings, Chu *et al*. firstly divided the natural history of HBV infection into three phases of immune tolerance, immune clearance, and residual integration (14), and then divided it into four phases of immune tolerance, immune clearance, inactive carrier status and reactivation (15). The latter four phases are the current widely used classification of hepatitis B natural history.

However, in recent years, some scholars have challenged the concept of “immune tolerance phase”, because HBV-specific T cell immune response and liver injury have been found in patients with chronic HBV infection in the so-called “immune tolerance phase” (16). In addition, the vast majority of newborns and infants inoculated with hepatitis B vaccine can produce antibodies, and the antibody titer is very high. These evidence all suggests that the so-called “immune tolerance phase” actually has an immune response. Therefore, there is no absolute immune tolerance state in the natural history of HBV chronic infection. Although there is no consensus on the above views, these new findings at least suggest that the so-called immune tolerance phase does not mean that HBV will not cause damage to the liver. It has been found that the host immune pressure plays the main role for viral mutation (17, 18). Recently, lilly Y *et al*. analyzed whole HBV genome NGS data from 97 treatment-naïve patients and found that the specific HBV variants associated with disease progression (cirrhosis and HCC) were common in immune tolerance stage. Therefore, they proposed that screening for specific viral variants and early antiviral therapy during this phase may help to reduce the risk of HCC (19). Because of these, more and more clinical experts are worried about the management strategy that patients in the so-called “immune tolerance phase” are just given follow-up but no recommendation of antiviral therapy (20).

### How to Define the Immune Tolerance Phase of HBV Infection?

The “immune tolerance phase” of HBV infection has been defined and described in many international CHB management guidelines (5–8). Although these guidelines have some differences in the definition of “immune tolerance phase” of HBV infection, they also have common characteristics, such as positive HBsAg (persistent positive for no less than 6 months), positive HBeAg, high level of HBV-DNA (inconsistent threshold), persistently normal ALT (inconsistent upper limit of normal value), as well as no obvious inflammation, necrosis and fibrosis in liver pathology.

At present, the inconsistent definition of immune tolerance phase in different guidelines is mainly manifested in the level of serum HBV DNA and ALT. Because of the controversy in serum HBV DNA and ALT levels, clinicians must pay attention to following details when judging a chronic HBV infection whether in the immune tolerance phase. Firstly, the guidelines require that patients' serum ALT level is persistently normal, rather than a certain cross-sectional serum ALT within the normal range. Therefore, in clinical practice, we need patients to provide reliable laboratory reports of dynamic serum ALT and comprehensively evaluate various potential factors that may cause ALT level fluctuations. Secondly, on the premise of meeting other conditions, the higher the serum HBV-DNA level, the more likely the patient will be in the immune tolerance period. Because only the very high serum HBV DNA level can accurately indicate the peaceful coexistence of the virus and the host.

Although there is no consensus on the lower limit of serum HBV-DNA in immune tolerance phase, it is agreed that the higher the better. And most clinical experts suggested that serum HBV-DNA in immune tolerance phase should be more than 10^7^~10^8^ IU/mL. Therefore, for those HBeAg-positive HBV patients whose serum HBV DNA level is not high and ALT level is within the normal range, we should be careful to judge whether they are really in the immune tolerance phase. In fact, when defining the immune tolerance phase in various international guidelines, it is mentioned that the evidence of liver histological changes must be available. Therefore, clinicians cannot make a hasty decision of the patient in immune tolerance phase in the absence of pathological results of liver tissue (21).

It is worth noting that although liver biopsy may help clinicians to understand the liver inflammation or fibrosis of patients, and judge whether patients are in the immune tolerance phase. Unfortunately, in the real world, it is impractical to determine whether patients have liver inflammation or fibrosis through regular dynamic liver biopsy. Therefore, clinicians did not take the pathological results of liver tissue as an essential index to judge whether patients are immune tolerance phase in the actual diagnosis and treatment process of hepatitis B. This is also one of the important reasons why there are different opinions on whether antiviral therapy should be recommended in patients with chronic HBV infection with normal ALT and high HBV DNA level. Therefore, there is an urgent need for new laboratory indicators or convenient tools to help clinicians better evaluate the changes of liver histology. However, in the absence of liver biopsy, clinicians should not make a hasty judgment of the immune tolerance phase at present.

### Clinical Outcome of HBV Infection in Immune Tolerance Phase

Previous studies have shown that patients in immune tolerance phase have no inflammation in the liver, and their serum ALT level is in the normal range, so they do not need antiviral therapy, and the risk of HCC is relatively low (22, 23). However, some studies have reported that a large proportion of chronic HBV infected patients with normal ALT level and high HBV DNA level have potential liver inflammation (24–26), and the risk of cirrhosis and HCC in these patients is significantly increased in the future.

The limitation of immune partition of natural history of hepatitis B may be an important reason for this embarrassing reality of hepatic histological abnormalities in immune tolerance phase (20). Some scholars recommend no antiviral therapy is based on correct diagnosis of “real immune tolerance” patients. Only paying attention to or emphasizing positive HBeAg, normal ALT and high level HBV-DNA will expand the range of patients in “real immune tolerance” phrase. In fact, there are so-called “gray areas” in both the current immune tolerance phase and inactive phase (27). Patients with chronic HBV infection who are older and/or whose serum HBV DNA level is not particularly high are often not really immune tolerant (21, 27). The development and deterioration of disease observed at present may come from this “gray area” patients in the immune tolerance phase. Thus, patients with normal ALT in the “gray areas” should be paid more attention.

## Does ALT in Normal Range Mean Healthy Liver?

### The Function of ALT and the Origin of Its Normal Range

Serum ALT is a kind of intracellular functional enzyme catalyzing amino transfer reaction, which is mainly distributed in the liver. It is one of the most sensitive indicators to measure liver function and reflect liver damage (9). In normal condition, as long as a small amount of ALT is released into the blood, the activity and activity of the enzyme in the serum can be significantly increased. The concentration of ALT in hepatocytes was 1,000~3,000 times higher than that in serum. As long as 1% of hepatocytes are necrotic and the activity of enzymes in blood is doubled, thus serum ALT is a sensitive marker of acute hepatocyte injury (9).

Currently, the internationally accepted “normal” serum ALT level is generally less than 40 U/L. After years of observation on a large number of blood donors in 2002, some scholars proposed to modify the normal range of serum ALT, and the reference range of serum ALT should be adjusted to 0 ~ 30 U/L for men and 0~19 U/L for women, excluding various interference factors such as overweight, drugs, alcohol and virus infection (28). In 2006, experts in the field of liver disease from the United States clearly suggested that the reference range of ALT should be adjusted to 0~30U/ L for men and 0~19 U/L for women (29). In 2018, the updated AASLD guideline suggested that the reference level of serum ALT should be adjusted to <35U/L for men and <25U/ L for women (7). According to the EASL guidelines in 2017, the upper limit of normal serum ALT was lowered by 10 U/L, and serum ALT is considered normal only when its level is lower than 30 U/L (for both men and women) (6).

In fact, serum ALT detection equipment and reagents are different in different countries and regions, thus the results of normal range are not the same (30). The standardization of ALT normal range needs to be solved urgently. And the “individualized ALT reference level” may be also a new direction in the future.

### Relationship Between ALT and Liver Injury

The elevation of serum ALT level in different degrees indicates that there is inflammatory reaction in liver tissue. In the recovery period of the liver disease, with the gradual disappearance of inflammation, ALT levels will gradually return to normal. CHB is a chronic infectious disease which is mediated by HBV and infiltrated by a variety of inflammatory cells in the liver. For example, CTL induces apoptosis of target cells by secreting perforin and expressing FasL; Neutrophils and monocytes in the liver produce reactive oxygen species, carbon monoxide, and reactive nitrogen species, which mediate the injury of infected hepatocytes and the formation of local inflammatory injury and reaction with monocytes/macrophages infiltration. Generally, ALT in peripheral blood of patients with HBV infection leading to liver inflammatory injury will increase in varying degrees, but there are also some patients whose ALT in peripheral blood is still in the normal range or only slightly increased even when liver histology has shown more significant inflammation or fibrosis.

According to the histopathological analysis of 346 patients with chronic HBV infection (including 88 patients with ALT ≥ 2 × up limit of normal [ULN] and 258 patients with ALT <2 × ULN), 48.8% of them had obvious hepatic tissue inflammation (G ≥ 2). Among ALT ≥ 2 × ULN group, 68.2% of patients had obvious liver inflammation, while 42.2% of patients in ALT <2 × ULN group had obvious liver inflammation. We previously analyzed of the pathological results of 228 CHB patients with ALT <2 × ULN and found that 49.2% of patients had significant inflammation (G ≥ 2) and 36.4% of patients had significant fibrosis or cirrhosis (S ≥ 2) (26).

It can be seen that serum ALT level and liver inflammation degree are not always parallel, and low level of ALT does not necessarily mean that there is no inflammation or mild inflammation in liver tissue. In fact, chronic HBV infections with normal or slightly elevated ALT levels may have entered the immune clearance period. After hepatocyte injury, ALT released into the blood will soon lose its activity and not remain at a high level. Therefore, it is necessary to dynamically monitor the serum ALT level to evaluate the liver inflammation. We can't judge whether there is inflammation in the liver simply according to whether the serum ALT level is normal or not.

### CHB With Normal ALT Still Had Obvious Liver Histological Abnormalities

We previously analyzed the pathological results of 141 CHB patients with normal ALT and found that 47.5% of the patients had significant inflammation and 33.3% had significant fibrosis or cirrhosis (26). Among HBeAg-positive patients with persistently normal ALT, the proportion of patients with significant liver inflammation or fibrosis was 27.8~49.4% (31–34). Recently, Prof. Zhuang and his colleagues also reported that 53.2% of HBeAg-negative patients with normal ALT have obvious liver fibrosis (35). Among them, 44.6% of patients with serum ALT >20 U/L had obvious hepatic necrotizing inflammation and 61.0% had obvious hepatic fibrosis; while only 26.5% of patients with ALT ≤ 20 U/L had obvious hepatic necrotizing inflammation and 41.7% had obvious hepatic fibrosis. These results confirmed that a considerable number of HBeAg-negative CHB patients with normal ALT had obvious liver histopathological changes, and even 46.2% of the patients with low HBV DNA level (<2,000 IU/mL) had obvious liver fibrosis. A long-term follow-up study of 1,965 untreated inactive carrier HBeAg-negative patients in Taiwan found that during an average follow-up of 11.5 years, 16% progressed to reactivation and 3% developed cirrhosis (36). Therefore, patients with normal ALT in inactive carrier status may also have significant liver pathological changes and high risk of liver cancer or death (10, 36, 37).

In some patients with chronic HBV infection, liver inflammation is not serious, ALT level is normal, but liver fibrosis is very obvious. Clinical evidences have shown that serum ALT level is in the normal range for a long time but still could occult progression to cirrhosis, or even HCC (11). Therefore, although serum ALT is a sensitive indicator of liver inflammation, it cannot reflect all the pathological changes of liver tissue, especially the degree of liver fibrosis and its progression. For patients with obvious liver histological changes but low serum level of ALT, the higher ULN of ALT may be a possible reason for this phenomenon.

## Benefits of Antiviral Therapy in Patients With Normal ALT

It has long been recognized that timely and effective antiviral treatment can significantly reduce the risk of end-stage liver disease and related death events in CHB patients. In the past, patients with normal ALT level in the clinical immune tolerance period have not advocated antiviral therapy, because they are worried that after antiviral therapy, not only the viral DNA is not effectively suppressed, but it may induce HBV drug resistance mutations (5–8). In addition, many experts believe that for HBV-infected people with normal ALT in the immune tolerance phase, the virus and the host are in a state of mutual balance, and the virus generally will not cause obvious damage to the host liver. Moreover, whether direct antiviral therapy in patients with immune tolerance will affect the spontaneous immune clearance remains unknown, and the economic burden of patients also increase. Therefore, they infer that these patients may not benefit significantly from antiviral treatment. However, by establishing Markov model, Kim *et al*. found that compared with delaying antiviral treatment to active hepatitis stage, the former can reduce the risk of cirrhosis and HCC (38). Whether or not the patients with immune tolerance are supported to receive antiviral therapy at this stage; and no matter what stage of the natural history of CHB patients, antiviral therapy can delay the progress of the disease, improve the survival time and quality of life.

In recent years, some scholars have carried out in-depth analysis on the relationship between HBV DNA level and the risk of cirrhosis and HCC. Patients with extremely high HBV DNA level may be really in immune tolerance phase. For those patients with normal ALT, it is reasonable not to recommend antiviral therapy. On the contrary, for the patients whose HBV DNA level is not very high but ALT level is normal, antiviral therapy should be considered (27). In addition, due to the limitations of previous detection techniques, HBV DNA in peripheral blood cannot be accurately quantified. However, with the increase of sensitivity of detection techniques, the persistent low-level viremia (LLV) and the risk of disease progression are also concerned (39, 40). It is believed that LLV cannot only increase the risk of drug resistance but also increase the risk of cirrhosis and HCC in the future. In fact, the LLV patients include three groups, namely, naïve patients with low viral load (HBeAg negative chronic HBV infection with normal ALT), nucleos(t)ide analogs-treated patients with low viral load (receiving antiviral treatment, but the HBV DNA in peripheral blood is still detectable) and the patients with low HBV DNA level after drug withdrawal. For these HBV-infected patients with normal ALT, effective antiviral therapy should not be ignored, although the relevant clinical evidence is not sufficient.

## Summary

In general, ALT has some limitations as an indication for initiating antiviral therapy and not all patients in so-called immune tolerance stage do not need treatment. Accurate evaluation of “real immune tolerance” is the key to the decision of treatment or not.

For patients in “real immune tolerance” period (positive HBeAg, normal ALT,HBV-DNA > 10^7^-10^8^ IU/ml, age <30 years, no or mild inflammation/fibrosis in the liver) and without extrahepatic diseases, antiviral treatment can be postponed for the low risk of liver disease progression, and the most important intervention are comprehensive professional follow-up and monitoring. Once the patient enters the immune clearance period, treatment must be initiated in time. For patients who cannot be clearly diagnosed, threatment can be provided with good communication. During the process of treatment, here are some recommendations for patients and physicians: (1) the first-line NAs (ETV,TDF, TAF) are the best choice; (2) instructions on medication use should be given to avoid patients' arbitrary discontinuation; (3) Serum HBV-DNA should be monitored for all patients on antiviral therapy, and prompt genotypic resistance testing and remedial treatment measures should be taken as early as possible, once increase of HBV-DNA level detected; (4) for eligible patients, combination or sequential PEG-IFN therapy should be provided to improve the clinical cure rate.

It is worth noting that when deciding to treat or not, the patient is the final decision maker while the physician is the advisor. The coexistence of risk and opportunity makes the occurrence of any small probability event a 100% fact for the patient. Physicians should objectively inform patients about the status quo, the pros and cons of antiviral therapy, and leave the decision to patients. Finally, controversies stem from the lack of high-quality information. It is more important for medical professionals to devote efforts to perform high-quality clinical and basic research, revise more reliable diagnostic strategies for immune tolerance phrase, and accelerate development of new drugs to relieve the dilemma of drug resistance and cure CHB eventually.

## Author Contributions

JZ, F-DW, M-LW, Y-CT, D-BW, Y-JS, G-BX, X-BC, and XC conceived this review and collected the literature, E-QC conducted the study supervision and revised the manuscript. All authors contributed to the article and approved the submitted version.

## Conflict of Interest

The authors declare that the research was conducted in the absence of any commercial or financial relationships that could be construed as a potential conflict of interest.

## Publisher's Note

All claims expressed in this article are solely those of the authors and do not necessarily represent those of their affiliated organizations, or those of the publisher, the editors and the reviewers. Any product that may be evaluated in this article, or claim that may be made by its manufacturer, is not guaranteed or endorsed by the publisher.
